# Establishment of age-related AMH screening cutoffs in Chinese women with PCOS: a retrospective study using propensity score matching analysis

**DOI:** 10.1186/s12902-025-01975-4

**Published:** 2025-07-01

**Authors:** Zhen Wang, Xiaojing Teng, Yanfei Liu, Yonghai Shen, Lin Ma, Yu Chen

**Affiliations:** 1 Department of Clinical Laboratory, Hangzhou Women’s Hospital (Hangzhou Maternity and Child Health Care Hospital), Hangzhou, China; 2 Department of Clinical Laboratory, Hangzhou First People’s Hospital, Hangzhou, China; 3 Department of Gynecology and Obstetrics, Hangzhou Women’s Hospital (Hangzhou Maternity and Child Health Care Hospital), Hangzhou, China

**Keywords:** Polycystic ovary syndrome, anti-Müllerian hormone, Propensity score matching, Follicle-stimulating hormone, Luteinizing hormone

## Abstract

**Background:**

Polycystic ovary syndrome (PCOS) is a common endocrine disorder that affects 5–18% of women worldwide. This disorder is characterized by two core pathological features: ovulatory dysfunction and hyperandrogenism.

**Method:**

A retrospective analysis was conducted on cases treated at Hangzhou Women’s Hospital from July 2021 to November 2023. Based on age, we conducted propensity score matching analysis in a 1:1 ratio and statistical analysis using SPSS software, plotted receiver operating characteristic curves, and obtained the area under the curve, the optimal anti-Müllerian hormone screening threshold, corresponding sensitivity, specificity, Youden index, correct diagnostic rate, positive predictive value, negative predictive value. Age-related anti-Müllerian hormone screening criteria with polycystic ovary syndrome were established and evaluated based on the optimal anti-Müllerian hormone screening cutoff for each group. The above results were validated by the validation group. And Kappa consistency test was conducted between the age-related anti-Müllerian hormone screening test and 2003 Rotterdam criteria for polycystic ovary syndrome.

**Results:**

The screening criteria for age-related anti-Müllerian hormone with polycystic ovary syndrome were: ≥7.46 ng/ml (20-24-year-old), ≥ 4.55 ng/ml (25-29-year-old), ≥ 4.19 ng/ml (30-34-year-old), ≥ 3.57 ng/ml (35-39-year-old).

**Conclusion:**

Our research indicates that age specific AMH threshold values show promising sensitivity and specificity for PCOS screening in the validation cohort. These findings indicate the potential utility of AMH based screening, but emphasize the need for optimization and validation in larger multicenter studies before clinical implementation.

**Clinical trial number:**

Not applicable.

## Introduction

Polycystic ovary syndrome (PCOS) is a common endocrine disorder that affects 5–18% of women worldwide [1], according to the Rotterdam diagnostic criteria. A 2013 epidemiological survey of 16,886 community women of childbearing age in 10 provinces and cities in China [2] showed that the prevalence of PCOS was 5.61%. Since then, epidemiological studies in various regions have found that the incidence rate of PCOS is on the rise. For example, in 2014, the reported prevalence of PCOS in Chengdu was 7.1% [3], and in 2019, 173 cases of PCOS were diagnosed in 1781 women aged 16 to 30 years old in Daxing District, Beijing, with a incidence rate of 9.71% [4]. In 2021, WU Q et al. [5] conducted a meta-analysis of 69 studies involving 154,599 participants, which showed that the latest PCOS prevalence rate in China has risen to 10.01%.The pathological features of this disease include reproductive axis dysfunction, metabolic disorders, and adrenal cortex dysfunction. Clinical manifestations include acne, hirsutism, infrequent or absent menstruation, all of which can lead to impaired ovarian function and reduced fertility [6].

Anti-Müllerian hormone (AMH) is a peptide growth factor [7]. It is found in the granulosa cells of primary, preantral, and small antral follicles with a diameter less than 8 mm [8]. AMH plays a role in regulating the recruitment, development, and selection of follicles [9, 10]. The research established a positive correlation between serum AMH levels and antral follicle count (AFC) [11]. AMH is secreted by granulosa cells of preantral and antral follicles, and its concentration directly reflects the number of remaining follicles in the ovary, making it one of the most sensitive indicators for evaluating ovarian reserve. The AMH level shows a decreasing trend with age, gradually decreasing after reaching its peak during puberty and approaching zero after menopause, which can predict the risk of fertility decline and premature ovarian failure. AMH is currently the best indicator for evaluating ovarian reserve function, and the reference range for AMH varies among women of different age groups [12]. AMH, as a diagnostic indicator for PCOS, can reduce misdiagnosis caused by subjective factors such as ultrasound examination, help to more accurately identify PCOS patients, and thus improve the targeting and effectiveness of treatment. We conducted a comprehensive evaluation of ovarian reserve function by detecting AMH levels in women of different age groups and combining them with other relevant indicators. Meanwhile, we also noticed the differences in AMH levels among different age groups. Therefore, when analyzing the data, we conducted stratified analysis on samples from different age groups to ensure the accuracy and reliability of the results. This study aims to establish age-related screening criteria for PCOS using AMH as an indicator.

## Materials and methods

### Patients

Study design and participant selection: This retrospective cohort investigation analyzed clinical data from female patients receiving care at Hangzhou Women’s Hospital over a 28-month period (July 2021 to November 2023). Patients are screened through the hospital’s electronic medical record system, which includes reproductive medicine center clinics (IVF-ET consultation), endocrine metabolism clinics, and general gynecological clinics, to ensure coverage of different treatment needs. The participant selection process involved systematic screening of electronic health records from outpatient services using the Rotterdam diagnostic criteria (2003) (13)for polycystic ovary syndrome (PCOS). Eligible cases were required to demonstrate ≥ 2 of the following clinical manifestations: (1) oligo-ovulation or anovulation, (2) biochemical or clinical hyperandrogenism, and/or (3) ultrasonographic evidence of polycystic ovarian morphology.

Data collection methodology: A comprehensive medical record evaluation protocol was implemented, incorporating four key diagnostic components: detailed reproductive history documentation, standardized physical examinations, endocrine laboratory profiling (including serum androgen measurements), and transvaginal ultrasonographic assessments. This multi-modal approach enabled cross-verification of diagnostic parameters and clinical presentation alignment with PCOS diagnostic standards.

Exclusion criteria were strictly applied to ensure sample integrity. If an individual is under 20 years old or over 39 years old and has used sex hormones or other drugs (which have an impact or interference on the various hormones tested in this study) within the past 3 months before the test, they will be excluded. Those with diabetes, congenital adrenal hyperplasia, or other conditions that could lead to hyperandrogenism were also excluded. Hyperandrogenemia, a pathological state in the female endocrine system, is characterized by elevated androgen levels or activity in the serum or increased sensitivity of peripheral target organs to androgens. This can disrupt the hypothalamic-pituitary-ovarian axis and cause energy metabolism disorders. These conditions were identified through patient medical records and, when needed, additional laboratory tests. Furthermore, individuals with acute or chronic infections, systemic inflammatory diseases, or incomplete clinical data were excluded. The control group consisted only of women who did not exhibit any Rotterdam standard performance.

Data Collection: In this study, we compiled a comprehensive dataset that included detailed patient demographics and clinical parameters. The demographic data collected included age, weight, height, and Body Mass Index (BMI). Clinical histories were thoroughly reviewed, covering menstrual and reproductive histories, past medical conditions, and detailed family medical histories. Endocrine profiles were meticulously evaluated through a series of laboratory tests. These tests measured levels of follicle-stimulating hormone (FSH), luteinizing hormone (LH), prolactin (PRL), testosterone (T), the LH-to-FSH ratio, and anti-Müllerian hormone (AMH). All laboratory procedures followed international standard operating protocols within our hospital’s medical laboratory department to ensure the accuracy and reliability of the results. Our hospital uses Beckman Coulter DXI800 electrochemiluminescence analyzer for reproductive hormone testing, and the reagents are purchased from Haier Shi Biomedical Co., Ltd. The AMH detection adopts Roche E411 electrochemiluminescence analyzer, and the reagents are purchased from Roche Diagnostic Products (Shanghai) Co., Ltd.

### Statistics and analysis

Data processing and hypothesis testing were conducted using IBM SPSS Statistics v26.0. The study employed a comprehensive statistical approach to analyze the data. Measurement data were presented as median (Q1, Q3), while counting data were expressed as (N, %). The normality of the data was assessed using the Single Sample Kolmogorov-Smirnov test. For measurement data that followed a normal distribution, homogeneity of variance was first tested. If the variance was uniform, one-way ANOVA was applied; if not, Welch ANOVA was used. For non-normal distribution measurement data, the Mann-Whitney U test was employed, with *P* < 0.05 indicating statistical significance. Counting data were compared using chi-square test or Fisher’s exact test. Following the chi-square test, Bonferroni corrected z-test was used for column proportion comparison. All tests were two-tailed, with *P* < 0.05 considered statistically significant.

We constructed receiver operating characteristic (ROC) curves for AMH with and without incorporating the LH/FSH ratio as a screening test. From these curves, we derived several key metrics: Area Under the Curve (AUC): A measure of the overall diagnostic performance of the test, with higher values indicating better discrimination between disease and non-disease states. Optimal AMH Screening Cutoff Value: The threshold value of AMH that maximizes both sensitivity and specificity for distinguishing between PCOS and non-PCOS cases. Sensitivity: Calculated as (true positive)/(true positive + false negative), reflecting the test’s ability to correctly identify individuals with the disease. Specificity: Calculated as (true negative)/(true negative + false positive), indicating the test’s ability to correctly identify individuals without the disease. Youden Index: Defined as Sensitivity + Specificity − 1, ranging from 0 to 1. A value of 1 indicates perfect discrimination, while a value of 0 suggests the test performs no better than random guessing. Correct Diagnosis Rate (CDR): Calculated as (true positive + true negative)/total, representing the overall accuracy of the test. Positive Predictive Value (PPV): Calculated as (true positive)/(true positive + false positive), indicating the probability that a positive test result correctly identifies an individual with the disease. Negative Predictive Value (NPV): Calculated as (true negative)/(true negative + false negative), indicating the probability that a negative test result correctly identifies an individual without the disease. These metrics collectively provide a comprehensive evaluation of the diagnostic performance of AMH as a screening tool, both independently and in combination with the LH/FSH ratio.

We use DeLong test to determine if there is a statistical difference between the AUC values of two ROC curves. Set the significance level to 0.05, if *P* < 0.05, it is considered that there is a significant difference in the AUC values between the two ROC curves; If *P* ≥ 0.05, it is considered that there is no significant difference in AUC values between the two ROC curves. Kappa consistency test was conducted between the test group and the 2003 Rotterdam criteria. Kappa value < 0.20 indicates poor consistency; 0.21–0.40 indicates weak consistency; 0.41–0.60 indicates moderate consistency; 0.61–0.80 represents the consistency height; 0.81-1.00 indicates strong consistency. To validate the diagnostic thresholds for AMH in different age groups, we conducted a validation study. This involved evaluating the performance of the AMH threshold screening test in a separate validation group. Through this process, we obtained key metrics, including sensitivity, specificity, Youden index, correct diagnosis rate, positive likelihood ratio, and negative likelihood ratio. Additionally, a Kappa consistency test was performed to assess the agreement between the results of the validation group and the 2003 Rotterdam criteria, ensuring the reliability of the diagnostic thresholds established. To evaluate the correlation between AMH levels and age, we conducted a correlation test. Given that AMH and age may not follow a normal distribution in this study, we employed Spearman’s rank correlation test, which is suitable for non-parametric data. The significance level was set at *P* < 0.05. All analyses and visualizations were performed using R version 4.4.2.

According to the inclusion and exclusion criteria, a total of 346 cases of PCOS and 315 cases of non-PCOS were initially identified. To minimize potential bias caused by differences in baseline data, we performed propensity score matching analysis at a 1:1 ratio based on age. This resulted in a final test group consisting of 213 PCOS cases and 213 non-PCOS cases. Additionally, to validate the diagnostic thresholds, we re-collected cases and established an external validation group. This validation group was divided into a validation PCOS subgroup and a validation non-PCOS subgroup, each comprising 30 cases, for a total of 60 cases. This study was approved by the Ethics Committee of Hangzhou Women’s Hospital (Ethical Review Number: Medical Ethics Review A-070).

## Results

### Distribution of clinical characteristics and hormone levels of test group

No significant differences in age were observed between the two groups. However, the BMI, FSH, AMH level, LH, PRL, T, and LH/FSH ratio were all statistically significantly different (*P* < 0.05) (Table 1).

**Table 1:** Summary of clinical characteristics and hormone levels in PCOS and Non-PCOS

Variables	PCOS *n* = 213	Non-PCOS *n* = 213	Total *n* = 426	*P*-value	Validation-PCOS *n* = 30	Validation-non-PCOS *n* = 30	Total *n* = 60	*P*-value
Age (year)								
Median (Q1, Q3)	29.00(27.00,32.00)	29.00(27.00,32.00)	29.00(27.00,32.00)	1.000^a^	26.00(24.00,28.00)	26.00(24.00,28.00)	26.00(24.00,28.00)	1.000 ^a^
20–24	21(9.86)	21(9.86)	42(9.86)	1.000^b^	10(33.33)	10(33.33)	20(33.33)	1.000 ^b^
25–29	96(45.07)	96(45.07)	192(45.07)		18(60.00)	18(60.00)	36(60.00)	
30–34	76(35.68)	76(35.68)	152(35.68)		2(6.67)	2(6.67)	4(6.67)	
35–39	20(9.39)	20(9.39)	40(9.39)		0(0.00)	0(0.00)	0(0.00)	
BMI (kg/m^2^)								
Median (Q1, Q3)	22.04(19.54,24.57)	20.28(18.45,22.04)	20.82(19.00,23.34)	< 0.0001^a^	22.58(19.95,26.12)	21.37(17.72,24.20)	21.85(19.24,24.99)	0.059 ^a^
< 18.5	27(12.68)	57(26.76)	84(19.72)	< 0.0001^b^	2(6.67)	10(33.33)	12(20.00)	0.072 ^b^
18.5-<24	124(58.22)	129(60.56)	253(60.82)		15(50.00)	12(40.00)	27(45.00)	
24-<28	38(17.84)	21(9.86)	59(13.85)		7(23.33)	5(16.67)	12(20.00)	
≥ 28	24(11.27)	6(2.82)	30(7.21)		6(20.00)	3(10.00)	9(15.00)	
FSH (IU/L)								
Median (Q1, Q3)	6.30(5.52,7.72)	6.77(5.85,8.20)	6.59(5.61,7.91)	0.004 ^a^	5.77(4.97,7.36)	7.12(6.13,7.78)	6.63(5.26,7.64)	0.034 ^a^
LH (IU/L)								
Median (Q1, Q3)	11.04(5.86,15.64)	4.96(3.55,6.95)	6.51(4.17,12.25)	< 0.0001 ^a^	10.20(5.87,14.49)	5.67(4.29,11.04)	8.26(5.11,13.04)	0.013 ^a^
≤ 5.9	54(25.35)	134(62.91)	/	< 0.0001 ^b^	7(23.33)	16(53.33)	/	0.017 ^b^
>5.9	159(74.65)	79(37.09)	/		23(76.67)	14(46.67)	/	
PRL (ng/ml)								
Median (Q1, Q3)	10.79(7.78,13.75)	12.39(9.04,16.51)	11.58(8.44,14.94)	< 0.0001 ^a^	12.76(10.34,16.27)	12.99(9.58,17.28)	12.93(10.20,16.28)	0.894 ^a^
≤ 15.3	176(82.63)	150(70.42)	/	0.003 ^b^	21(70.00)	21(70.00)	/	1.000 ^b^
>15.3	37(17.37)	63(29.58)	/		9(30.00)	9(30.00)	/	
T (ng/ml)								
Median (Q1, Q3)	0.67(0.53,0.84)	0.44(0.35,0.58)	0.56(0.40,0.74)	< 0.0001 ^a^	0.87(0.74,0.94)	0.57(0.50,0.64)	0.66(0.56,0.85)	< 0.0001 ^a^
≤ 0.75	126(59.15)	199(93.43)	/	< 0.0001 ^b^	7(23.33)	29(96.67)	/	< 0.0001 ^b^
>0.75	87(40.85)	14(6.57)	/		23(76.67)	1(3.33)	/	
AMH (ng/ml)								
Median (Q1, Q3)	7.51(5.12,11.09)	3.57(2.25,5.18)	5.15(3.08,8.19)	< 0.0001 ^a^	8.00(5.62,10.42)	4.04(2.42,7.09)	6.07(3.56,8.98)	< 0.0001 ^a a^
< 3	16(7.51)	87(40.85)	/	< 0.0001 ^b^	2(6.67)	8(26.67)	/	0.006 ^b^
3-<5	35(16.43)	70(32.86)	/		3(10.00)	10(33.33)	/	
5-<9	82(38.50)	46(21.60)	/		13(43.33)	8(26.67)	/	
≥ 9	80(37.56)	10(4.69)	/		12(40.00)	4(13.33)	/	
LH/FSH ratio								
Median (Q1, Q3)	1.61(0.94,2.37)	0.69(0.51,1.04)	1.00(0.59,1.86)	< 0.0001 ^a^	1.81(1.11,2.47)	0.83(0.64,1.46)	1.30(0.78,1.97)	< 0.0001 ^a^
≤ 0.749	37(17.37)	119(55.87)	/	< 0.0001 ^b^	2(6.67)	11(36.67)	/	0.005 ^b^
> 0.749	176(82.63)	94(44.13)	/		28(93.33)	19(63.33)		

*Note:* PCOS = polycystic ovary syndrome; AMH = anti-Mullerian hormone; LH = luteinizing hormone; FSH = follicle-stimulating hormone; T = testosterone; ^a^ P-value were obtained using Mann–Whitney U-test; and ^b^ P were obtained using chi- square test

### Statistical analysis of various indicators from different age in test group

Age stratification was strictly aligned with the manufacturer-defined reference intervals of Roche cobas^®^ assay kits (REF No. 06331076190, Roche study number RD001727) [13], which categorize age groups as: 20–24, 25–29, 30–34, 35–39 years based on established population-specific biomarker variations. There were 21, 96, 76, and 20 cases respectively.

In 20-24-year-old group, age, BMI, FSH, LH, PRL, and LH/FSH ratio revealed no significant differences (*P* > 0.05). AMH levels and T differed statistically significant (*P* < 0.05) (Table 2).

**Table 2:** Summary of clinical characteristics and hormone levels from different age in test group

Variables	PCOS	Non-PCOS	*P*-value
20-24-year-old			
Age(year)	24.00(21.00,24.00)	24.00(21.00,24.00)	1.000
BMI (kg/m^2^)	20.57(18.28,23.23)	18.55(17.58,22.06)	0.170
FSH(IU/L)	5.66(4.87,6.97)	6.99(5.62,8.43)	0.058
LH(IU/L)	7.28(3.80,16.90)	6.06(4.88,9.14)	0.435
PRL (ng/ml)	12.60(8.64,14.83)	14.56(10.14,22.15)	0.128
T(ng/ml)	0.76(0.56,0.95)	0.57(0.42,0.69)	0.012
AMH (ng/ml)	7.97(5.42,12.17)	5.34(3.29,6.40)	0.003
LH/FSH ratio	1.25(0.67,2.33)	0.89(0.59,1.42)	0.090
25-29-year-old			
Age(year)	27.00(26.00,29.00)	27.00(26.00,29.00)	1.000
BMI (kg/m^2^)	21.91(19.53,23.78)	20.02(18.37,21.85)	< 0.0001
FSH(IU/L)	6.53(5.60,7.70)	6.57(5.83,7.88)	0.495
LH(IU/L)	11.17(6.95,15.52)	4.95(3.21,7.03)	< 0.0001
PRL (ng/ml)	11.45(8.67,14.24)	13.17(10.02,17.27)	0.004
T(ng/ml)	0.67(0.49,0.84)	0.47(0.36,0.58)	< 0.0001
AMH (ng/ml)	7.73(5.16,11.37)	3.66(2.40,5.02)	< 0.0001
LH/FSH ratio	1.69(1.05,2.38)	0.76(0.50,1.04)	< 0.0001
30-34-year-old			
Age (year)	32.00(31.00,33.00)	32.00(31.00,33.00)	1.000
BMI (kg/m^2^)	22.65(19.59,25.57)	20.31(18.82,22.19)	0.001
FSH(IU/L)	6.31(5.51,7.94)	7.08(5.91,8.51)	0.046
LH(IU/L)	10.96(5.98,14.99)	4.90(3.82,6.54)	< 0.0001
PRL (ng/ml)	9.93(7.10,13.40)	11.78(8.63,16.18)	0.028
T(ng/ml)	0.66(0.52,0.81)	0.42(0.31,0.57)	< 0.0001
AMH (ng/ml)	7.46(5.24,10.20)	3.65(2.08,5.13)	< 0.0001
LH/FSH ratio	1.55(0.94,2.24)	0.65(0.51,0.88)	< 0.0001
35-39-year-old			
Age(year)	37.00(35.00,38.00)	37.00(35.00,38.00)	1.000
BMI (kg/m^2^)	22.05(21.24,26.72)	20.68(19.66,23.15)	0.073
FSH(IU/L)	6.43(4.48,7.70)	7.54(5.79,8.78)	0.121
LH(IU/L)	9.82(3.61,17.90)	4.03(2.83,6.60)	0.001
PRL (ng/ml)	10.11(5.97,12.87)	10.41(8.30,14.74)	0.147
T(ng/ml)	0.81(0.61,1.08)	0.37(0.32,0.46)	< 0.0001
AMH (ng/ml)	5.70(3.24,7.61)	2.12(1.82,2.99)	< 0.0001
LH/FSH ratio	1.53(0.60,2.98)	0.56(0.42,0.77)	0.002

*Note:* PCOS = polycystic ovary syndrome; AMH = anti-Mullerian hormone; LH = luteinizing hormone; FSH = follicle-stimulating hormone; T = testosterone

In 25-29-year-old group, there were no significant differences in age and FSH between two groups (*P* > 0.05). However, significant differences were observed in BMI, LH, PRL, AMH levels, T, and LH/FSH ratio (*P* < 0.05) (Table 2).

In 30-34-year-old group, there was no significant difference in the age (*P* > 0.05). However, the distribution of BMI, FSH, LH, PRL, AMH levels, T, and LH/FSH ratio differed significantly between two groups (*P* < 0.05) (Table 2).

In 35-39-year-old group, there was no significant difference in the age, BMI, FSH, and PRL (*P* > 0.05). However, the distribution of LH, AMH levels, T, and LH/FSH ratio differed significantly between two groups (*P* < 0.05) (Table 2).

**Fig. 1:**
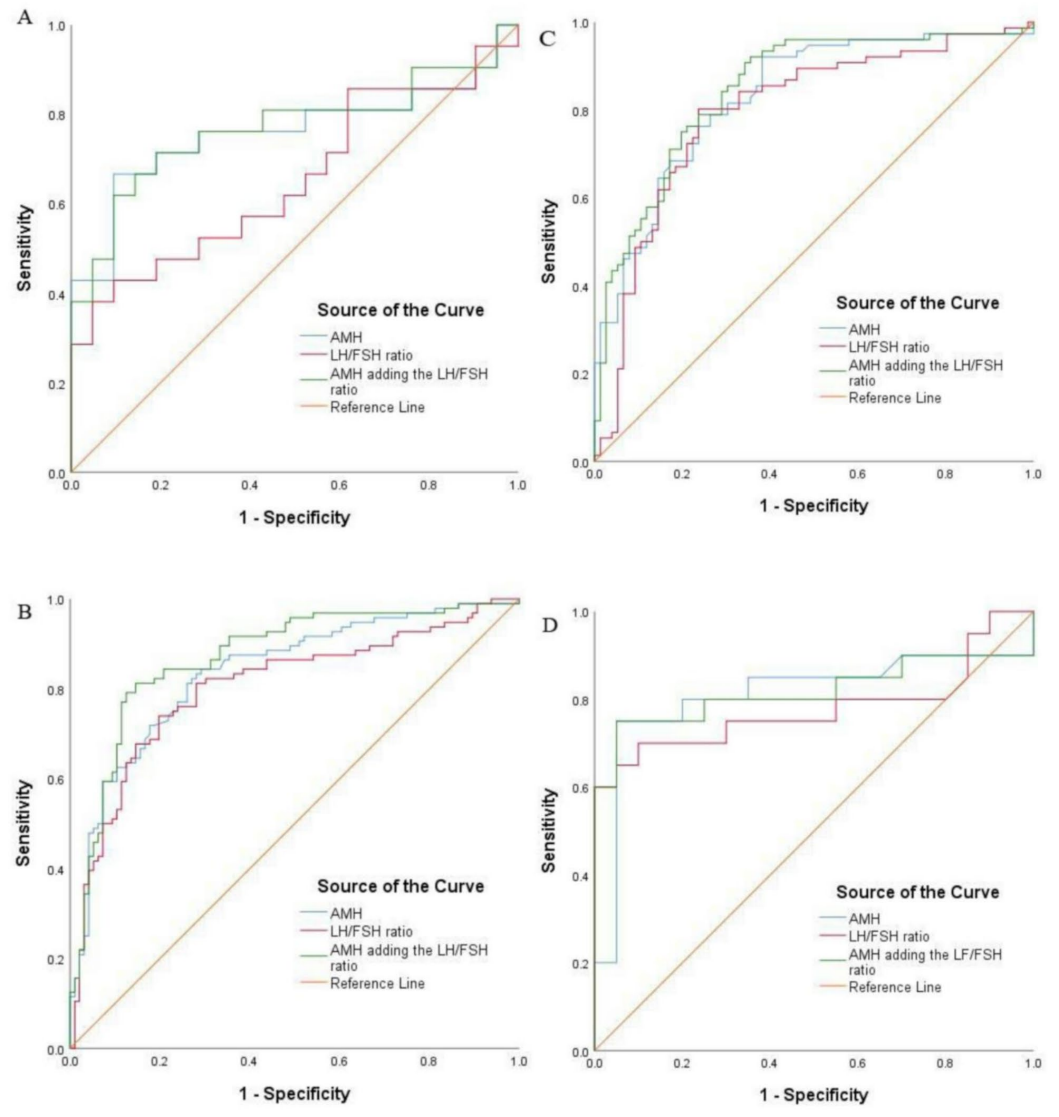
ROC curve of AMH adding or not the LH/FSH ratio screening for PCOS: **(A)** in 20-24-year-old **(B)** in 25-29-year-old **(C)** in 30-34-year-old **(D)** in 35-39-year-old

We used SPSS to draw ROC curves of AMH adding or not the LH/FSH ratio ​​for PCOS screening tests in test group (Fig. 1), and obtained the AUC of the ROC curve, the optimal AMH screening cutoff value, the corresponding sensitivity, specificity, Youden index, correct diagnosis rate, positive likelihood ratio, and negative likelihood ratio. There was no significant difference between two ROC curves for each age group(*P*>0.05) (Table 3). According to the age-related AMH screening test results in test group (Table 4), the sensitivity of this AMH screening criterion in test group was 0.84, a specificity of 0.73, a Youden index of 0.57, CDR 78.17%, PPV0.75, and NPV0.82.

**Table 3:** AUC of AMH adding or not the LH/FSH ratio ​​for PCOS screening tests in test group

	AUC	*P*-value	95% CI	sensitivity	specificity	Youden index	CDR	PPV	NPV
20-24-year-old									
AMH	0.76	0.529	0.60–0.92	0.67	0.91	0.57	78.60%	0.88	0.73
AMH adding the LH/FSH ratio	0.77		0.62–0.92	0.71	0.81	0.52	76.19%	0.79	0.74
25-29-year-old									
AMH	0.84	0.072	0.78–0.89	0.82	0.73	0.55	77.60%	0.75	0.80
AMH adding the LH/FSH ratio	0.87		0.82–0.92	0.79	0.88	0.67	83.33%	0.86	0.81
30-34-year-old									
AMH	0.83	0.250	0.77–0.90	0.92	0.62	0.54	76.97%	0.71	0.89
AMH adding the LH/FSH ratio	0.85		0.79–0.91	0.92	0.65	0.57	77.63%	0.73	0.88
35-39-year-old									
AMH	0.81	0.844	0.66–0.96	0.75	0.95	0.70	75.00%	0.94	0.79
AMH adding the LH/FSH ratio	0.82		0.67–0.97	0.75	0.95	0.70	75.00%	0.94	0.79

*Note:* AMH = anti-Mullerian hormone; LH = luteinizing hormone; FSH = follicle-stimulating hormone; AUC = Area under the curve; CI = confidence interval; CDR = correct diagnosis rate; PPV = positive predictive value; NPV = negative predictive value

**Table 4:** AMH screening test results for PCOS in test group and validation group

		2003 Rotterdam criteria for PCOS		Total
		PCOS	Non-PCOS	
Test group	positive	178	58	236
	negative	35	155	190
	total	213	213	426
Validation group	positive	24	11	35
	negative	6	19	25
	total	30	30	60

*Note:* PCOS = polycystic ovary syndrome

### Establishment of age-related AMH screening criteria for PCOS

Based on the aforementioned optimal AMH screening boundaries for PCOS in test group, the screening criteria for age-related AMH with PCOS were: ≥7.46 ng/ml (20-24-year-old), ≥ 4.55 ng/ml (25-29-year-old), ≥ 4.19 ng/ml (30-34-year-old), ≥ 3.57 ng/ml (35-39-year-old).

A Kappa consistency test was conducted between the AMH screening test and 2003 Rotterdam criteria, resulting in a Kappa value of 0.56(*P* < 0.01). The age-related AMH screening test results for PCOS in test group were presented (Table 4).

### Validation of age-related AMH screening criteria for PCOS

A comparison was made of the distribution of clinical characteristics and hormone levels between the validation-PCOS and the validation-non-PCOS. Within the validation of two groups, no significant differences were observed in age, BMI, and PRL (*P* > 0.05). However, the distribution of FSH, LH, AMH, T, and LH/FSH ratio were found to be significantly different(*P* < 0.05) (Table 1).

### Validation results of AMH screening criteria for PCOS

The AMH screening test results for PCOS in validation group were presented (Table 1). According to this table, the sensitivity was 0.80, the specificity 0.63, the Youden index 0.43, CDR 71.67%, PPV 0.69, and NPV0.76. The Kappa consistency test was performed between the validation group and the original PCOS diagnosis results. The Kappa value was 0.43(*P* < 0.01).

### Correlation analysis between AMH and age

**Fig. 2:**
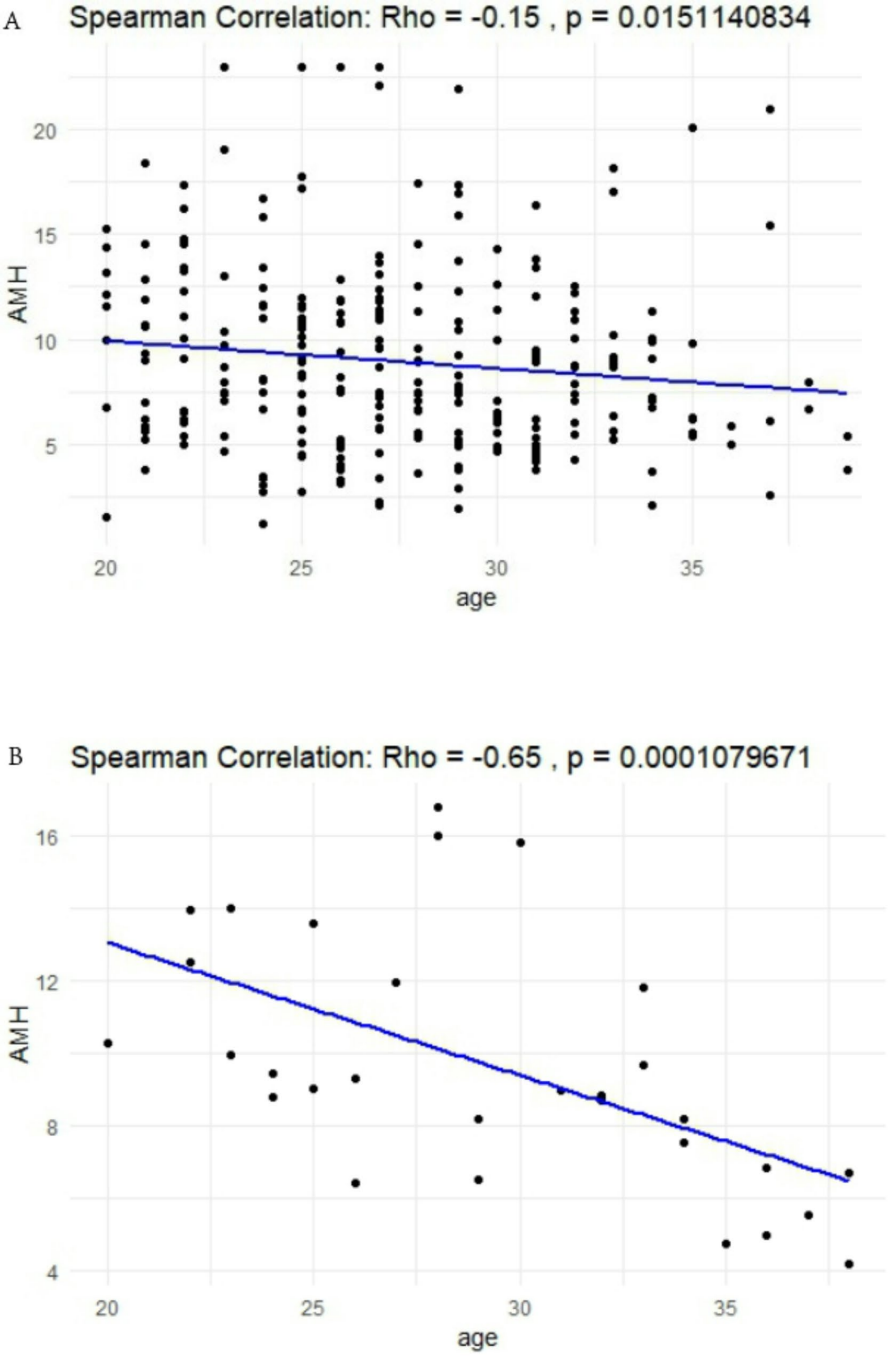
Correlation analysis between AMH and age: **(A)** in test group the correlation analysis between AMH and age **(B)** in validation group the correlation analysis between AMH and age

In the PCOS group of the test group, there was a significant negative correlation between AMH and age (*r*=-0.15, *p* < 0.05) (Fig. 2A). In the PCOS group of the validation group, we obtained consistent correlations: AMH and age (*r*=-0.65, *p* < 0.05) (Fig. 2B).

## Discussion

Polycystic ovary syndrome (PCOS) is an endocrine disorder characterized by hyperandrogenism, ovulation disorders, and metabolic abnormalities. Patients often seek medical attention for infertility or menstrual disorders, and their ovarian pathological manifestations include thinning of the granulosa cell layer and accelerated follicular atresia, which are directly related to elevated androgen levels [14–16]. In addition, 40–60% of patients were associated with obesity and insulin resistance, and the risk of diabetes and cardiovascular disease was significantly increased [17, 18]. This disease also causes more than 10% of patients to experience endometrial hyperplasia and mental health problems (such as depression/anxiety) before the age of 40 [19].The internationally recognized diagnostic standards mainly include the National Institutes of Health (1992) and Rotterdam criteria [20]. They are all based on European and American ethnic groups and are not completely suitable for Chinese women.

Anti-Mullerian hormone (AMH), as an important biomarker of ovarian reserve, can be influenced by various factors. Age is a key factor affecting AMH levels. As age increases, AMH levels typically decrease, reflecting a reduction in ovarian reserve [21]. Additionally, studies have shown significant differences in AMH levels among different races and ethnicities. For example, African American women may have lower AMH levels at a younger age, but their decline may not be as significant with age, whereas Latin American and Chinese women may have lower AMH levels across all age groups compared to white women [22]. Smoking is also a significant factor affecting AMH levels. Smoking is associated with the decline in ovarian function and may reduce AMH levels by impacting ovarian reserve [23]. Additionally, medication use can also affect AMH levels. For example, certain chemotherapy drugs have been shown to cause damage to ovarian function, thereby affecting the recovery of AMH levels and the assessment of ovarian reserve [24].

The serum AMH level is an indicator of the number of follicles growing, reflecting the state of androgens within the follicles [25], possibly due to the early stages of follicle growth stimulated by androgens [26, 27]. AMH levels also independently reflect the size of the follicular (“ovarian reserve”) pool [28]. The levels of serum androgens and testosterone are positively correlated with AMH levels [29]. There is a strong correlation between AMH concentration and the number of antral follicles, AMH may be a useful biomarker for follicular pool and ovarian aging [30, 31]. Serum AMH levels remain stable throughout different periods of the menstrual cycle with no significant difference [32–34], the differences between the same individuals during the menstrual cycle are minimal [35]. Many scholars suggest that AMH should be used as the diagnostic criteria for PCOS or as a supplement to the Rotterdam PCOS diagnostic criteria [36, 37]. The application of AMH in the medical field mainly includes the following aspects: 1. AMH level shows a decreasing trend with age, gradually decreasing after reaching its peak during puberty and approaching zero after menopause, which can predict the risk of fertility decline and premature ovarian failure. Therefore, serum AMH levels may be correlated with the severity of PCOS symptoms (hyperandrogenism or ovulation disorders)0.2. In assisted reproductive technology, AMH levels are used to assess a woman’s potential fertility and help predict ovarian responsiveness to assisted reproductive technology (ART). 3.In most cases, serum AMH is correlated with the number of oocytes obtained after exogenous gonadotropin controlled ovarian stimulation. Therefore, serum AMH is a reliable predictor of ovarian response to controlled ovarian stimulation [38, 39]. 4.AMH can predict ovarian response to ovulation inducing drugs and help develop personalized ovulation induction plans. Low AMH indicates a low risk of ovarian response and requires an increase in the dosage of ovulation promoting drugs; High AMH signals the risk of ovarian hyperstimulation syndrome (OHSS) and requires adjustments to the treatment plan to reduce complications.5.AMH therapy has been proven effective in the field of tumor fertility to prevent the loss of primordial follicles observed during chemotherapy treatment, particularly through exposure to alkylating agents [40]. 6.AMH is a specific circulating indicator of granulosa cell tumors, with a diagnostic sensitivity of 76–91% and specificity of 90–100% [41]. The serum AMH level is directly proportional to the size of ovarian granulosa cell tumors and has a direct correlation with imaging results [42–44]. The new guidelines for PCOS in 2023 still use the Rotterdam criteria, but explicitly state that serum AMH testing can be used instead of pelvic ultrasound for the diagnosis of adult PCOS [45]. The concentration of AMH is closely related to clinical, endocrine, and ultrasound indicators, and may be a diagnostic biomarker for PCOS.

Anti-Müllerian hormone (AMH) trends in the Chinese population can be influenced by several factors, including genetic, environmental, and lifestyle differences. A study examining the distribution of serum AMH levels among infertile Asian women, including Chinese participants, found that age, ethnicity, obesity, and polycystic ovarian syndrome (PCOS) significantly impacted serum AMH levels. The study highlighted that the rate of AMH decrease accelerated with age [46].

Reviewing previous studies, we found that serum AMH levels in PCOS were 2–4 times higher than those in normal [47], and serum AMH levels in patients with only polycystic ovary changes were significantly higher than those in normal, but significantly lower than those in PCOS [48]. In vivo and in vitro animal experiments [49] have confirmed that AMH may be involved in follicle development, indicating that AMH may inhibit the initiation of follicle growth. There is evidence that AMH counteracts the effects of FSH on aromatase activity, follicular growth and development [50, 51].

The screening criteria for age-related anti-Müllerian hormone (AMH) and polycystic ovary syndrome (PCOS) have significant implications for Chinese women of childbearing age. 1.Improving the accuracy of PCOS diagnosis: Adopting age-related AMH screening criteria can more accurately identify PCOS patients and improve the accuracy and timeliness of diagnosis. 2.Optimizing the allocation of medical resources: Through more accurate diagnosis and monitoring of treatment effectiveness, medical resources can be allocated reasonably, misdiagnosis and unnecessary treatment can be reduced, and medical efficiency can be improved. 3.Promoting women’s health: The application of age-related AMH screening criteria can help detect and intervene in PCOS early, improve patients’ quality of life and reproductive health, and reduce the risk of long-term complications such as metabolic syndrome and cardiovascular disease.

In this study, the AUC of the ROC curves in test group were greater than 0.7(*P* < 0.001), indicating that the age-related AMH screening test has high prediction accuracy. In the validation part, there is an imbalance between the sensitivity (80%) and specificity (63%) of AMH threshold, which may lead to an increase in clinical false positive rate. This phenomenon is related to insufficient age stratification, confounding factors (such as obesity) interference, indicating limitations in the universality of a single threshold. Although the Youden index and Kappa consistency did not reach the ideal level, the negative predictive value of AMH still has clinical reference value when excluding non PCOS cases, especially for the initial screening of high-risk populations.

Although this study focused on the combined diagnostic efficacy of AMH and its LH/FSH ratio, as shown in Fig. 1, the use of LH/FSH ratio alone demonstrated certain diagnostic potential in the 20–24 age group (AUC = 0.65, *p* = 0.09, 95% CI 0.48–0.82) and 35–39 age group (AUC = 0.78, *p* = 0.03, 95% CI 0.62–0.96).

This study did not include individuals aged ≤ 19 and ≥ 40, which may limit the generalizability of the results. Future research should expand the age range to validate the generalizability of conclusions.

## Data Availability

All data generated from this study are available from the corresponding author upon reasonable request.
